# Mitigating role of financial inclusion on the perceived difficulties, concerns, and borrowing for medical expenses in Saudi Arabia

**DOI:** 10.1186/s12962-023-00506-z

**Published:** 2024-01-09

**Authors:** Mohammed Khaled Al-Hanawi, Naseem Al Rahahleh

**Affiliations:** 1 Department of Health Services and Hospitals Administration, Faculty of Economics and Administration, King Abdulaziz University, Jeddah, Saudi Arabia; 2 Health Economics Research Group, King Abdulaziz University, Jeddah, Saudi Arabia; 3 Finance Department, Faculty of Economics and Administration, King Abdulaziz University, Jeddah, Saudi Arabia

**Keywords:** Financial inclusion, Financial hardship, Healthcare, Medical expenses, Saudi Arabia

## Abstract

Access to convenient quality healthcare at all times is considered a basic human right; however, many countries are still striving to achieve this goal for their populations. The persistent rise in healthcare expenditure remains a significant obstacle in achieving universal health coverage on a global scale. The aim of this study was to investigate the role of financial inclusion in addressing the financial hardship related to health and medical expense concerns in the Kingdom of Saudi Arabia. Probit models were applied to analyse nationally representative data from the Global Financial Inclusion (Global Findex) database. The results showed that financial inclusion had a significant impact on reducing the hardship associated with obtaining money for emergency expenses within 30 days as indicated by a significant coefficient of -0.262. Additionally, Financial inclusion substantially increases the likelihood of borrowing money for health or medical purposes in the past 12 months, with a coefficient of 0.585. Moreover, correlations were identified between low income levels and decreased likelihood of borrowing for health/medical purposes, increased difficulty in obtaining money for emergency expenses, and heightened concern regarding the ability to afford medical costs in the event of serious illness or accidents. These findings highlight the need for policy makers and health providers to prioritize financial inclusion and support programs for low-income individuals to achieve equity in health treatment for all in Saudi Arabia.

## Introduction

The persistent rise in healthcare expenditure remains a significant obstacle in achieving universal health coverage (UHC) on a global scale. Health expenses are responsible for impoverishing almost 100 million individuals annually worldwide [1]. A critical goal for healthcare systems is to foster fairness and sustainability by decreasing per capita healthcare costs, particularly among marginalized communities [2]. Evidence indicates that a significant proportion of out-of-pocket expenditures for healthcare has the potential to trigger coping mechanisms (such as borrowing and selling assets) among households, which may have adverse long-term implications [3]. To address this issue, UHC was embraced as a core element of the Sustainable Development Goals (SDGs) agenda adopted by the United Nations in 2015 [4]. Nevertheless, healthcare expenditure remains a considerable burden for certain populations in both developing and developed nations [5].

Financial inclusion is recognised as a crucial element in achieving Sustainable Development Goals (SDG). Precisely, in SDG number 3, which focuses on health and well-being, given its clear influence on household health expenses [6]. Financial inclusion pertains to the capacity of individuals and households to obtain and utilize available financial services, including bank accounts, credit, and insurance [7]. The concept encompasses all efforts that make formal financial services accessible and affordable, particularly to low-income populations. Higher financial inclusion creates opportunities to improve financial stability over time through a more comprehensive and diversified financial system [8]. Previous studies in this field demonstrated that increased financial inclusion substantially reduces poverty and income inequality rates [9, 10]. The COVID-19 pandemic has accelerated digital financial inclusion, turning it into a potent tool for enhancing financial access and offering assistance to individuals and households during and after the pandemic [11, 12]. However, there is scarce literature addressing the impact of financial inclusion on the financial hardship related to health and medical expense concerns.

Paying for health services and medicine out-of-pocket (OOP) can cause substantial financial hardship for of individuals and households. Excessive OOP payment for healthcare costs can lead to societal impoverishment, prompting countries to bolster financial protection and ease the burden on government resources worldwide [13]. Financial inclusion in health enhances healthcare access for individuals and households. While prior studies have explored different aspects of financial inclusion, the healthcare aspect remains understudied. These studies have demonstrated that financial inclusion reduces poverty and inequality rates [8], significantly contributes to human development [14], improves the welfare of women [15], enhances economic development [16], and reduces the incidence of depressive symptoms [9]. The few studies that have explored the relationship between financial inclusion and healthcare have primarily employed a limited definition of financial inclusion [7], focused on specific health aspects [17], examined the issue only in low-income countries [10], or employed non-econometric techniques [18]. Therefore, there is a need for further research to investigate how financial inclusion relates to various aspects of healthcare financial hardship and their associations with relevant socio-economic characteristics.

Accordingly, the aim of this study was to address the existing gaps in research on the relationship between financial inclusion and healthcare financial hardship indicators. Based on the latest Global Financial Inclusion (Global Findex) dataset for 2021 [19], an expanded multidimensional financial inclusion index was constructed, and a probit model—as an econometrically based technique—was used to perform a rigorous analysis of the impact of financial inclusion and demographic factors on various financial hardship indicators related to health and medical expenses in the Kingdom of Saudi Arabia (KSA). This approach enabled consideration of multiple dimensions of healthcare expenses, specifically examining the effect of financial inclusion on borrowing for health or medical purposes in the past 12 months, perceived difficulty in obtaining money for emergency expenses within 30 days, perceived difficulty in obtaining money for emergency expenses within 7 days, and perceived worry regarding the inability to afford medical costs in case of an emergency. These results can therefore offer insights into the multifaceted relationship between financial inclusion and financial hardship in the KSA.

There are several compelling reasons to consider the KSA as a case study. Firstly, the KSA’s publicly funded healthcare system, which relies heavily on oil revenues and provides healthcare services to citizens free of charge at the point of use, is experiencing increased strain due to rising expenses and oil market volatility [20]. These current challenges, coupled with the system’s significant share of the national budget, offer an interesting model to examine how financial inclusion could enhance a healthcare system’s long-term sustainability. Secondly, a previous decomposition analysis of out-of-pocket health expenditure disparities in the KSA revealed that the financial burden disproportionately affects impoverished individuals [5]. This finding underscores the potential impact of financial exclusion on healthcare access inequalities, highlighting the role financial inclusion may play in mitigating disparities. Furthermore, despite prioritization by the KSA government in developing healthcare services at all levels and registering significant improvements in population health, the healthcare system continues to face numerous challenges, including a shortage of local healthcare professionals, changing disease patterns, and high demand for free services [21]. Given these circumstances, the results of this study are expected to inform policy makers in the KSA, and other countries with similar characteristics, on how to design and implement effective strategies to improve access to healthcare services by enhancing financial inclusion and reducing financial hardship among various demographic groups.

## Materials and methods

### Data

The primary data for this study were collected from the World Bank Global Findex database for the year 2021 [19]. This database provides financial information for more than 120 countries, including the KSA, through a survey. In particular, the Global Findex serves as the sole comprehensive global demand-side dataset, enabling in-depth analysis on a global and regional scale. These data thus offer valuable insights into how adults worldwide engage in saving, borrowing, payment transactions, and managing financial risks. The data collected for the Global Findex 2021 edition were derived from surveys conducted in more than 120 economies, ensuring national representation and encompassing approximately 128,000 adults. This latest edition builds upon its predecessors from 2011, 2014, and 2017. Notably, the 2021 version introduced new indicators that gauge financial health and resilience, offering the opportunity to obtain a more nuanced understanding of financial health. Furthermore, the 2021 edition presents more detailed data on digital payment adoption, including information on merchant and government payments.

Adaptations were required in the data collection process for the Global Findex survey in the KSA during 2021 due to the COVID-19 pandemic. Traditionally, face-to-face interviews were conducted for data collection; however, mobility restrictions posed challenges in some economies, including the KSA, in 2021. As a result, phone-based surveys, including calls to both mobile phones and landlines, were carried out in the KSA during the pandemic. The respondent selection procedure for phone-based economies, including the KSA, followed a similar approach to that adopted in previous years: random digit dialling or a nationally representative list of phone numbers was used to select respondents.

The dataset used in this study comprised 1019 respondents from the KSA. However, the data from 12 respondents were excluded due to missing responses and data from an additional 11 respondents were removed because they did not provide complete answers to the relevant questions related to variables of interest. Hence, our analysis was limited to respondents who had completed information on all the variables of interest, resulting in a final sample of 996 respondents.

### Variables

Four outcome variables were used to measure different aspects of financial hardship in the data relevant to the KSA. All variables were set as dummy variables (with values assigned 0 or 1) to enable binary analysis in the models. The first variable addressed borrowing money for health or medical purposes in the past 12 months (1 if the individual indicated that they had borrowed money for health/medical purposes and 0 otherwise). This variable provides insights into the financial burden of healthcare for individuals and families. The second variable was the perceived difficulty in obtaining a specified amount of money within 30 days for emergency expenses (1 if the individual indicated difficulty in reserving emergency funds in the next 30 days and 0 otherwise), reflecting the availability of financial resources for emergency or unexpected expenses. The third variable was the perceived difficulty in obtaining a specified amount of money within 7 days for emergency expenses (1 if the individual indicated difficulty in reserving emergency funds in the next 7 days and 0 otherwise). The final variable considered in this analysis was the perceived worry regarding the inability to afford medical costs in case of an emergency such as a serious illness or accident (1 if the individual had a level of worry and 0 otherwise), providing a measure of the need for financial support for health and medical expenses and the overall level of financial security for individuals and families.

The independent variables used in this analysis, as guided by previous studies [7, 22, 23], were financial inclusion, gender, age, education, and income. Financial inclusion has been defined as possessing a bank account, having taken a loan from a financial institution, or having savings in a financial institution [7]. This definition was expanded for the context of the present study by including the criterion of having an ATM/debit card connected to a financial institution as an additional form of financial inclusion. This addition recognizes the critical role that ATM/debit cards play in providing individuals with greater access to financial services and enabling them to participate more fully in the formal financial sector. The presence or absence of financial inclusion was recorded as a dummy variable, taking a value of 1 if the individual met any of the criteria mentioned above and 0 otherwise. Categorizing the level of financial inclusion in this manner could provide valuable insights into the extent to which individuals are participating in the formal financial sector.

Gender was coded as 1 for males and 0 for females. Age was categorized into four groups: 15–25 years (reference group), 26–35 years, 36–44 years, and ≥ 45 years. Education level was collected in the survey through three categories: primary education (0 to 8 years), secondary education (9 to 15 years), and tertiary education (over 15 years, with a college certificate or higher). These categories were used to display descriptive information, while dummy variables set for each education level were used in the probit models. The lowest education level, primary education, was used as a reference group in the sample analysis. The survey collected income information through five quintiles, ranging from the poorest to the richest. Income quintiles were used for descriptive purposes in our analysis and the categories were converted to dummy variables for each quintile for probit model analysis. The poorest income level (quintile 1) was used as the reference group.

### Estimation modelling

An estimation model was constructed to examine the influence of financial inclusion and demographic characteristics on several financial hardship indicators (outcome variables, *Y*) related to health and medical expenses, including borrowing for health or medical purposes in the past 12 months (*Y1*), perceived difficulty in obtaining money for emergency expenses within 30 days (*Y2*), perceived difficulty in obtaining money for emergency expenses within 7 days (*Y3*), and perceived worry regarding the inability to afford medical costs in case of an emergency (*Y4*).

Our estimation model was guided by the study of Al-Hanawi et al. [7], which suggested that financial hardship faced by individuals is determined by their ability to borrow for medical purposes. Our model also controlled for personal attributes such as gender, age, education, and income, based previous research demonstrating that these factors play a role in determining financial hardship for health/medical purposes [7, 22].

The general estimation equations for the outcome variables were as follows:

*Y1*_*i*_ = *α*_*1*_ + *α*_*2*_ (financial inclusion)_*i*_ + *α*_*3*_*X*_*i*_ + *µ*_*i*_ *(1)*

*Y2*_*i*_ = *α*_*1*_ + *α*_*2*_ (financial inclusion)_*i*_ + *α*_*3*_*X*_*i*_ + *µ*_*i*_ *(2)*

*Y3*_*i*_ = *α*_*1*_ + *α*_*2*_ (financial inclusion)_*i*_ + *α*_*3*_*X*_*i*_ + *µ*_*i*_ *(3)*

*Y*4_*i*_ = *α*_*1*_ + *α*_*2*_ (financial inclusion)_*i*_ + *α*_*3*_*X*_*i*_ + *µ*_*i*_ *(4)*

*X* denotes control variables that represent personal attributes. The error term (*µi*) was assumed to be independently distributed.

The equations were estimated using a simple probit regression model based on maximum-likelihood estimation and default standard errors. In fact, the probit model serves as a tool for modelling binary or dichotomous outcome variables. Within the probit model, the probability’s inverse standard normal distribution is represented as a linear combination of predictor variables [24].

Before estimating the model, we examined the correlation matrix for the predictors and found that none of the correlations exceeded the 0.9 cut-off proposed by Pallant [25], indicating that there were no serious collinearity issues. All analyses were conducted using STATA software (StataCorp LP, Texas, USA) and statistical significance was judged at the 5% level.

## Results

### Descriptive analysis

Table 1 shows the distribution of the study characteristics in groups of respondents categorized as financially included (individual met any of the criteria for financial inclusion) and non-financially included (none of the criteria met); the majority of the sample (n = 824, 82.7%) was considered to be financially included, while 172 (17.3%) were non-financially included. Among the total 996 respondents, 53.5% were female and 46.5% were male. With regard to the age distribution, 47.2% of the sample were in the range of 26 to 35 years old, while 6.2% were 45 years old or older. Regarding education level, 49.9% of the sample had completed tertiary education, 48.7% had completed secondary education, and only 1.4% had primary education as their highest education level. The sample was representative of all the targeted income quintiles, with the poorest quintile represented by 13.6%, second quintile by 16.2%, middle quintile by 18.1%, fourth quintile by 22.8%, and the richest quintile by 29.4% of the total sample. Among the financially included, the highest levels of financial inclusion were observed among women, individuals aged 26 to 35 years, and those with tertiary education. Furthermore, a positive association was found between income quintiles and financial inclusion, indicating that as income levels increased, the level of financial inclusion also increased.

**Table 1:** Characteristics of the study population (n = 996)

Characteristics	Categories	Non-financially included		Financially included		Total	
		N	%	N	%	N	%
Gender	Female	56	32.6	477	57.9	533	53.5
	Male	116	67.4	347	42.1	463	46.5
Age	15–25 years	61	35.5	266	32.3	327	32.8
	26–35 years	75	43.6	395	47.9	470	47.2
	36–44 years	25	14.5	112	13.6	137	13.8
	≥ 45 years	11	6.4	51	6.2	62	6.2
Education	Primary education	2	1.2	12	1.5	14	1.4
	Secondary education	109	63.4	376	45.6	485	48.7
	Tertiary education	61	35.5	436	52.9	497	49.9
Income quintiles	Poorest quintile	46	26.7	89	10.8	135	13.6
	Second quintile	41	23.8	120	14.6	161	16.2
	Middle quintile	25	14.5	155	18.8	180	18.1
	Fourth quintile	28	16.3	199	24.2	227	22.8
	Richest quintile	32	18.6	261	31.7	293	29.4
Total		172	17.3	824	82.7	996	100

### Levels of financial hardship across sample attributes

Table 2 presents the financial hardship levels across different sample attributes. Individuals who were financially included had greater financial hardship needs compared to those who were not financially included. Among the financially included sample, the most significant concern regarding health funds was the perceived difficulty in accessing money for emergency expenses within a 7-day period.

**Table 2:** Levels of financial hardship across sample attributes (n = 996)

Characteristics		Model 1		Model 2		Model 3		Model 4	
		Borrowing for health or medical purposes in the past 12 months		Perceived difficulty in obtaining money for emergency expenses within 30 days		Perceived difficulty in obtaining money for emergency expenses within 7 days		Perceived worry regarding the inability to afford medical costs in case of an emergency	
		No, N (%)	Yes, N (%)	No, N (%)	Yes, N (%)	No, N (%)	Yes, N (%)	No, N (%)	Yes, N (%)
Financially included									
	No	157 (18.9)	15 (9.1)	49 (10.9)	123 (22.6)	28 (10.3)	144 (19.9)	73 (16.2)	99 (18.2)
	Yes	674 (81.1)	150 (90.9)	402 (89.9)	422 (77.4)	245 (89.7)	579 (80.1)	379 (83.8)	445 (81.8)
Gender									
	Female	442 (53.2)	91 (55.2)	273 (60.5)	260 (47.7)	178 (65.2)	355 (49.1)	256 (56.6)	277 (50.9)
	Male	389 (46.8)	74 (44.8)	178 (39.5)	285 (52.3)	95 (34.8)	368 (50.9)	196 (43.4)	267 (49.1)
Age (years)									
	15–25	271 (32.6)	56 (33.9)	128 (28.4)	199 (36.5)	69 (25.3)	258 (35.7)	143 (31.6)	184 (33.8)
	26–35	400 (48.1)	70 (42.4)	238 (52.8)	232 (42.6)	144 (52.7)	326 (45.1)	233 (51.5)	237 (43.6)
	36–44	112 (13.5)	25 (15.2)	61 (13.5)	76 (13.9)	44 (16.1)	93 (12.9)	57 (12.6)	80 (14.7)
	≥ 45	48 (5.8)	14 (8.5)	24 (5.3)	38 (7.0)	16 (5.9)	46 (6.4)	19 (4.2)	43 (7.9)
Education									
	Primary	13 (1.6)	1 (0.6)	3 (0.7)	11 (2.0)	1 (0.4)	13 (1.8)	4 (0.9)	10 (1.8)
	Secondary	387 (46.6)	98 (59.4)	183 (40.6)	302 (55.4)	102 (37.4)	383 (53.0)	201 (44.5)	284 (52.2)
	Tertiary	431 (51.9)	66 (40.0)	265 (58.8)	232 (42.6)	170 (62.3)	327 (45.2)	247 (54.6)	250 (46.0)
Income quintile									
	Poorest	107 (12.9)	28 (17.0)	27 (6.0)	108 (19.8)	15 (5.5)	120 (16.6)	37 (8.2)	98 (18.0)
	Second	130 (15.6)	31 (18.8)	55 (12.2)	106 (19.4)	32 (11.7)	129 (17.8)	63 (13.9)	98 (18.0)
	Middle	155 (18.7)	25 (15.2)	73 (16.2)	107 (19.6)	40 (14.7)	140 (19.4)	74 (16.4)	106 (19.5)
	Fourth	198 (23.8)	29 (17.6)	106 (23.5)	121 (22.2)	62 (22.7)	165 (22.8)	102 (22.6)	125 (23.0)
	Richest	241 (29.0)	52 (31.5)	190 (42.1)	103 (18.9)	124 (18.9)	169 (23.4)	176 (38.9)	117 (21.5)
Total (N)		831	165	451	545	273	723	452	544

As shown in Tables 2 and 165 of the 996 total respondents have borrowed money for health or medical reasons in the last 12 months, with a slightly higher number of females (n = 91) who borrowed money compared to males (n = 74). Moreover, males encountered a higher level of difficulty in obtaining money for emergency expenses compared to females within both the 7-day and 30-day time frames.

Analysis of financial hardship revealed different patterns across different age groups. Respondents aged 26–35 years had the highest number of individuals who borrowed for health or medical purposes (70 out of 165), while the lowest number was observed among those aged ≥ 45 years. The age group of 26–35 years also faced the most challenges in terms of the perceived difficulty in obtaining money for emergency expenses within 7 and 30 days and the perceived worry regarding the inability to afford medical costs in case of an emergency. Therefore, the age group of 26–35 years encounters the most significant financial hardships. Notably, individuals aged ≥ 45 years generally exhibit lower levels of financial hardship in comparison.

An examination of financial hardship across different education groups also revealed notable patterns. Among the respondents, those with secondary education accounted for the highest number of individuals who borrowed for health or medical purposes (98 out of 165). Concerning the perceived difficulty in obtaining money for emergency expenses within 7 and 30 days, the secondary education group faced the most significant challenges, followed by the tertiary education group. Interestingly, both the secondary and tertiary education groups exhibited higher levels of perceived worry regarding the inability to afford medical costs in case of an emergency. In contrast, respondents with primary education displayed the least concerns across all key issues. Finally, among the income quintiles, the richest quintile exhibited the lowest number of individuals who perceive difficulty in obtaining money for emergency expenses within 30 days.

### Econometric analyses

Table 3 presents the results of econometric models, with all models demonstrating statistical significance (Prob > χ^2^) at the 0.05 level. The model examining perceived difficulty in obtaining money for emergency expenses within 30 days exhibited the highest variance coefficient, with a Pseudo R^2^ value of 9.66%.

**Table 3:** Results of probit regression models (n = 996)

Variable	Model 1			Model 2			Model 3			Model 4		
	Borrowing for health or medical purposes in the past 12 months			Perceived difficulty in obtaining money for emergency expenses within 30 days			Perceived difficulty in obtaining money for emergency expenses within 7 days			Perceived worry regarding the inability to afford medical costs in case of an emergency		
	Coeff.	*P*-value	Marginal	Coeff.	*P*-value	Marginal	Coeff.	*P*-value	Marginal	Coeff.	*P*-value	Marginal
**Financial inclusion**	0.585	< 0.001	0.139	-0.262	0.026	-0.093	-0.149	0.259	-0.045	0.104	0.357	0.039
**Gender**												
Female	Ref			Ref			Ref			Ref		
Male	0.026	0.793	0.006	0.337	< 0.001	0.119	0.402	< 0.001	0.122	0.189	0.025	0.071
**Age**												
15–25 years	Ref			Ref			Ref			Ref		
26–35 years	0.017	0.882	0.004	-0.124	0.207	-0.044	-0.123	0.245	-0.037	-0.046	0.625	-0.017
36–44 years	0.117	0.451	0.028	-0.189	0.166	-0.067	-0.372	0.011	-0.113	0.020	0.880	0.007
≥ 45 years	0.298	0.143	0.071	-0.062	0.740	-0.022	-0.225	0.256	-0.068	0.302	0.108	0.114
**Education**												
Primary education	Ref			Ref			Ref			Ref		
Secondary education	0.874	0.092	0.209	-0.217	0.587	-0.077	-0.462	0.368	-0.140	0.007	0.983	0.002
Tertiary education	0.534	0.305	0.128	-0.421	0.293	-0.149	-0.689	0.179	-0.209	-0.095	0.796	-0.036
**Income quintile**												
Poorest	Ref			Ref			Ref			Ref		
Second	-0.087	0.614	-0.020	-0.396	0.015	-0.140	-0.355	0.057	-0.107	-0.312	0.043	-0.118
Middle	-0.333	0.060	-0.079	-0.491	0.002	-0.174	-0.376	0.041	-0.114	-0.348	0.023	-0.131
Fourth	-0.385	0.026	-0.092	-0.693	< 0.001	-0.246	-0.612	0.001	-0.185	-0.456	0.002	-0.172
Richest	-0.112	0.489	-0.026	-1.140	< 0.001	-0.404	-0.990	< 0.001	-0.300	-0.823	< 0.001	-0.311
**Constant**	-2.049	< 0.001		1.245	0.003		1.846	0.001		0.451	0.247	
LR X^2^ (11)	34.79			132.57			103.07			58.68		
Prob > X^2^	0.0003			0.000			0.000			0.000		
Pseudo R^2^	0.0389			0.096			0.088			0.042		
Log likelihood	-429.7			-619.6			-533.4			-656.8		

Based on the model 1 results shown in Table 3, two exploratory variables had a significant impact on borrowing money for health or medical purposes in the past 12 months. Financial inclusion demonstrated a significant positive influence, with a coefficient of 0.585 and marginal effect of 0.139 at a probability of < 0.001. This indicates that individuals who are financially included have a higher likelihood of borrowing for health/medical purposes. Additionally, fourth-quintile income level showed a significant negative influence, with a coefficient of -0.385 and a marginal effect of -0.092 at a probability of 0.026. This suggests that as individuals move up in income quintile, the likelihood of borrowing for health/medical purposes decreases.

The findings from model 2 revealed that several exploratory variables had a significant impact on the hardship of acquiring money for emergency expenses within the next 30 days (Table 3). Financial inclusion emerged as a significant factor, with a coefficient of -0.262 and a marginal effect of -0.093 at a probability of 0.026. This indicates that an increase in financial inclusion is associated with a decrease in the hardship of obtaining money for emergency expenses within the specified timeframe, highlighting the crucial role of financial inclusion for individuals. Moreover, male gender was found to have a positive and significant influence, with a coefficient of 0.337 and a marginal effect of 0.119 at a probability of < 0.001. This suggests that males face a higher likelihood of experiencing difficulty in obtaining money for emergency expenses within 30 days compared to females. Additionally, all income quintiles demonstrated significant negative influences with a more negative influence with increasing income quintile. This implies that as income increases, the hardship of acquiring money for emergency expenses within 30 days diminishes.

The results of model 3 (Table 3) showed that gender plays a significant role in influencing the perceived difficulty in obtaining money for emergency expenses within 7 days, with a coefficient of 0.402 and a marginal effect of 0.122 at a probability of < 0.001. This suggests that males are more likely to experience hardship in obtaining money for emergency expenses within the next 7 days compared to females. Age also emerged as a significant predictor, with those aged 36–44 years showing a negative coefficient of -0.372 and a marginal effect of -0.113 at a probability of 0.011. This implies that as individual age increases, the perceived hardship in acquiring money for emergency expenses within 7 days decreases. Furthermore, all income quintiles demonstrated significant negative influences, with a more negative influence as income quintiles rise. This implies that higher income levels are associated with reduced perceived hardship in obtaining emergency funds within 7 days.

Finally, the findings from model 4 (Table 3) indicated that gender had a significant positive influence on perceived worry regarding the inability to afford medical costs in case of an emergency, with a coefficient of 0.189 and a marginal effect of 0.071 at a probability of 0.025. This suggests that males exhibit higher levels of worry compared to females. Additionally, all income quintiles demonstrated significant negative influences. This implies that as income levels increase, worries regarding the inability to afford medical costs in case of an emergency are reduced. These findings are therefore consistent with the earlier model results.

## Discussion

This study investigated how financial inclusion and demographic factors impact financial hardship faced by individuals in the KSA. The study deviates from the previous related literature by examining the influence of financial inclusion and demographic characteristics on several financial hardship indicators related to health and medical expenses, including borrowing for health or medical purposes in the past 12 months, perceived difficulty in obtaining money for emergency expenses within 30 days, perceived difficulty in obtaining money for emergency expenses within 7 days, and perceived worry regarding the inability to afford medical costs in case of an emergency. Additionally, a comprehensive multidimensional financial inclusion index was created in this study, utilizing the latest Global Findex dataset for 2021 and an econometric technique (the probit model) was applied to ensure the robustness of the findings. The results are relevant to the development and execution of financial inclusion strategies in the KSA.

In contrast to the findings of Shabir and Ali [18], who observed that financial product ownership and usage were higher among males than females in 2017, this study supports that for the data related to the year 2021, female participants had a higher level of financial inclusion (477 out of 533) than male participants (347 out of 463). This implies that financial inclusion measures may be more accessible and beneficial for women. Among the financially included, we observed lower financial inclusion levels among older age groups, specifically those between the ages of 36 to 44 years and those aged 45 years and above. In other words, the proportion of financially included individuals in these age groups is smaller compared to that in the younger age groups (15–25 years and 26–35 years) for the analysed dataset. As financial technology services continue to evolve, it is likely that younger generations will find it easier to participate compared to the older generation, who are more accustomed to manual structures [26].

Among individuals who have attained financial inclusion, those with higher levels of education consistently demonstrated the highest degrees of financial inclusion, aligning with findings from prior research [27, 28]. Education equips individuals with the necessary knowledge and resources to engage in financial activities, highlighting the need for the government to create an enabling environment for the banking sector to promote financial literacy among the less educated and impoverished population [29]. Furthermore, the present research findings confirm that financial inclusion is directly proportional to the income level of participants, in line with the findings of previous studies [30, 31]. This can be attributed to the fact that individuals with higher financial resources are better equipped to own the products used as indicators of financial inclusion and have the prerequisite knowledge to participate in the economy.

Overall, having perceived difficulty in obtaining money for emergency expenses within 7 days varied across all demographic characteristics. Male respondents not only encountered more difficulties in acquiring funds for emergency expenses within one week but also exhibited greater concern about all financial aspects pertaining to health and medical expenses compared to their female counterparts. In other words, male individuals have a higher possibility in experiencing hardship to obtain money for emergency expenses in the next 7 days than females. This may be attributed to cultural and social norms due to societal expectations and gender roles that place greater emphasis on males as the primary breadwinners or decision-makers regarding financial affairs. This can lead to men feeling more pressure to be financially responsible and concerned about their financial well-being [32, 33]. Similarly, as individuals’ age increases, the perceived hardship in acquiring money for emergency expenses within 7 days decreases. In fact, our results showed that as age increased, the level of concern gradually decreased. This can be attributed to the fact that the older generation is likely to have accumulated financial capacity and savings over time, which would lessen their worry compared to that of individuals in the younger generation who are still in the process of building their economic status [10, 15].

Participants in the higher income quintiles reported less concerns about meeting health and medical expenses compared to those in the lower income quintile, consistent with the findings reported in the literature [34–36]. Healthcare expenses tend to be higher among the poor, leading to higher levels of concerns and depressive symptoms. Therefore, it is crucial for the government to implement policies aimed at improving the education and income levels of marginalized populations.

In addition to using descriptive statistics, this study employed the probit model to determine the marginal impact of financial inclusion on financial hardship related to health and medical expenses. The results revealed that individuals who are financially included have a higher likelihood of borrowing for health and medical purposes. This finding is consistent with previous studies, which emphasize that an increase in financial inclusion is associated with higher household health expenditures [10, 16]. Financial inclusion improves people’s access to loan facilities. However, as found in this study, increases in income levels enhance people’s financial status and reduce the need for borrowing for health and medical purposes.

The study identified financial inclusion as a significant factor in the perceived difficulty in obtaining money for emergency expenses within 30 days, with a coefficient of -0.262 and a marginal effect of -0.093. This means that as financial inclusion increases, the perceived difficulty of having money available for emergency expenses within 30 days decreases. This finding is consistent with the impact of financial inclusion on the perceived difficulty of having money for emergency expenses within 7 days, which also decreases as financial inclusion increases. Enhancing access to financial services through financial inclusion is likely to reduce poverty and enhance human development [37, 38]. The improved financial capacity that comes with financial inclusion likely increases the ability of individuals to access money for emergency expenses within a short period of time.

Finally, this study revealed that financial inclusion did not significantly impact the perceived worry regarding the inability to afford medical costs in case of an emergency. Instead, the results revealed that gender plays a significant role as a determinant of perceived worry regarding the inability to afford medical costs in case of an emergency. Moreover, gender was found to have a significant positive influence, suggesting that men have a higher level of worry regarding the inability to afford medical costs in case of an emergency compared to women. Unlike women, men are regarded as breadwinners and assume the responsibility to ensure that healthcare expenses are covered. This makes them prone to tendencies of worry regarding the inability to pay for such services during an emergency [8]. Nonetheless, this finding is encouraging for the healthcare system, as the existence of excessive worry among women can lead to depressive tendencies, which can result in further health issues and put additional pressure on the already overburdened healthcare system [18]. Therefore, it is crucial that the government prioritizes gender-inclusive policies to promote equal empowerment and create a more inclusive society.

The study is not without limitations. Firstly, there is a possibility of sample selection bias since the study is based on data from the Global Findex dataset for 2021, which may not be representative of the entire population of the KSA. The dataset may only include individuals who have access to financial services or have participated in financial transactions, which may not cover individuals who are financially excluded. Secondly, the study is reliant on self-reported data, which may be subject to recall bias or social desirability bias. Participants may overestimate their level of financial inclusion or underestimate their healthcare expenditure to avoid social stigma or judgement. Moreover, the study could not establish a causal relationship between financial inclusion and healthcare expenditure, and other factors such as cultural or social factors could also play a role. Therefore, caution must be exercised in interpreting the results. Further research can explore a combination of both quantitative and qualitative data techniques to provide a more nuanced understanding of the relationship between financial inclusion and healthcare expenditure.

## Conclusion

This study examined the relationship between financial inclusion, demographic factors, and financial hardship indicators related to health and medical expenses in the KSA, using a multidimensional financial inclusion index and the latest Global Findex dataset for 2021. The probit model was employed to analyse the data. The results showed that financial inclusion had a positive impact on the ability to borrow and have money available for emergency expenses across various demographic characteristics. While respondents of all income categories reported difficulty in securing money for emergency expenses within 7 days and 30 days, this difficulty decreased with higher income levels.

These findings have important policy implications for addressing rising healthcare costs and for ensuring the effectiveness of a UHC policy. Governments should therefore focus on creating an enabling environment for the private sector to innovate and expand financial services, particularly in impoverished communities. This would reduce the cost of accessing financial services and increase a household’s capacity to spend more on healthcare needs. Additionally, efforts should be made to improve infrastructure such as roads, electricity, and information and communication technology to enhance access to finance for healthcare services. Overall, this study’s policy insights are valuable in supporting the KSA’s Vision 2030 through the promotion of a gender inclusion growth strategy.

## Data Availability

The datasets are available on the World Bank website. Available from: https://microdata.worldbank.org/index.php/catalog/4700/get-microdata.
